# Correspondence

**Published:** 1883-12

**Authors:** 


### Correspondence.

No. 1513 Walnut Street,
Philadelphia, Nov. 15th, 1883.

*Editor Independent Practitioner:*

First, let me congratulate you upon the success achieved by the Independent Practitioner, and send the management a little substantial aid. I know that it takes money to run a dental journal, and I doubt not you find many more ready to receive and read your journal than they are to pay the annual subscription. As an evidence of my appreciation of your excellent work, accept the enclosed check for ten dollars.

I have read with much interest your communication on "The condition of the teeth of certain pre-historic races," and your conclusions are so at variance with my former belief, that it makes me wonder why the teeth of the pre-historic man of North America should show so much disease, while those of the ancient Egyptian indicate so little. It was my pleasure some years ago to spend considerable time in Egypt and the Holy Land. While in Egypt I had very fine opportunities for studying the teeth of the mummied remains of the early people of that country. It was during the Franco-Prussian war. The gentleman who had charge of the mummy pits at Sakkarah was a Frenchman. He had gone home on a visit, and was locked up in Paris during the siege. A little money judiciously expended, made it possible for me to gain access to the mummy pits, and I spent a day there most delightfully. In these pits were thousands of mummies that had been there at least three, and probably four thousand years, and to all appearance the hand of man had never since touched them. With the aid of two Egyptian guides I pulled them out, unwound the linen bandages from the head, and examined the teeth. I cannot remember how many I took out—but dozens of them at least—and examined hundreds of others which had already been taken out and unwound. Instead of finding caries of the teeth, contracted jaws, or irregularities, I found splendid teeth, large, well-formed jaws, and with one or two trifling exceptions no irregularity in the position of the teeth in the arch.

I did find, however, great abrasion upon the masticating surfaces of the bicuspids and molars. This I could readily account for in the fact that the ancient Egyptians (the modern as well) were in the habit of eating food which required a great deal of masticating. I continued my investigations in the museums of Cairo and Alexandria, and I find the same excellent condition of the teeth in all of the mummies there.

At the Convent of St. Katherine, on Mt. Sinai, which was established in the third century, and which contains the skulls of all the monks who have died there in fifteen hundred years, I found good teeth in all of those who lived in the earlier centuries.

I came away from the East with the opinion pretty well fixed in my mind that the earlier inhabitants of that part of world had little or no need of dentists. Had I the time, and did space permit, I would like to tell you how beautifully the mummies in Egypt were embalmed. I have in my possession one of the skulls, several jaws, and a hand, which I brought with me when I returned.

Your paper has upset many of the theories which I have entertained with reference to the antiquity of dental diseases. I cannot believe that the same number of Egyptian skulls, taken from those who inhabited earth at the same period, would show a similiar condition. I did not find tartar in a single instance. In one case I did find a single tooth missing, and absorption which would indicate an abscess (perhaps chronic).

I shall look with interest for further communications upon the same subject.

Yours very truly,

EDWIN T. DARBY.

---

PALAZZO MAROTTI, 172 VIA NAZIONALE,
ROME, ITALY, Oct. 30th, 1883.

*Editor Independent Practitioner:*

I was very much pleased with your article in the October number upon "Conditions of the teeth of certain pre-historic American races." I am especially glad, because I am working on the same line myself, in examining into the teeth of the Etruscan people, a race which was old when the ancient Romans began their wonderful career.

Last May, I was requested by Prof. Helbig, of the German Archaeological Society of Rome, to make a report on my investigations at Corneto Tarquinius, which I hope to have ready by January next.

I expect to have good drawings made of two partial sets of teeth inserted on gold, which were taken from an Egyptian tomb, dating back five hundred years before the Christian era. I found evidences of nearly all the dental lesions which now torment the human race.

For several years I have been skeptical on the theories, certainties, and infallible proofs of our professional authorities, who were feeling their way in the twilight of doubtful evidence, and therefore I decided, if possible, to "go back of the returns" and see for myself, little dreaming that any one on your side of old ocean was doing likewise.

I am satisfied that your conclusions are correct, and that dental diseases are as old as the world, and not a modern new departure. As I am yet in "the woods" of investigation, I will not crow, but I feel that no amalgam "new departure" can compare in results with the departure you have made.

In the meanwhile, believe me sincerely yours,

J. G. VAN MARTER, A. B.; D. D. S.

---

No. 548 West 17th Street,
Philadelphia, Nov. 15th, 1883.

*Editor Independent Practitioner:*

In the report of the meeting of the Pennsylvania State Dental Society, in the Independent Practitioner for November, in the matter relating to the resolutions against dentists who invent and sell materials the formulas for which they deem it best not to publish, many of your readers may be led to believe that there is a law in that society against patentees of dental appliances. There is no such law. The article referred to reads as follows :

"ART. 7, SEC. 2. Any dentist or professor who shall procure a *patent for a remedy* or who shall enter into a collusive agreement with an apothecary to receive pecuniary compensation for patronage for sending his prescriptions to said apothecary, or who shall hereafter give a certificate in favor of a *patent remedy*, shall be disqualified from becoming or remaining an active member of any local society entitled to representation."

The American Dental Association is entitled to the full respect of the whole profession in having no such Puritanical laws in operation against *patentees of any kind.*

In speaking of such measures the late Professor T. L. Buckingham gave as his opinion that we have no right to make restrictive regulations—and the words expressing that opinion were the last that this respected and beloved man spoke in the Odontological Society of Pennsylvania.

Dental societies should in every way encourage all respectable practitioners to become members. Proscriptive laws of whatsoever kind should be rescinded, and their further introduction be made impossible. They are antagonistic to the spirit of our American institutions, tend to interfere with our progress, savor of tyranny, communism and bigotry, and are a reflection upon the intelligence of dentists. They are aimed against a very useful set of men in the profession, without whom there would be no real progress. They do the societies no good, because such men will not be *forced* to give away the result of their labors (hard work, which has extended over months and sometimes years,) to *spongers* who are unwilling to delve themselves. They are too apt to be introduced to gratify personal spleen, or to punish a less influential brother whose creed may be objectionable to a few in power.

My intimate acquaintance during the past twenty years with the Dental Society men of Philadelphia, has satisfied me that a very large majority of them have no sympathy with such regulations, and as recent experience has proven, are ready to expunge them *in toto* from our societies here.

